# A case of *Aspergillus* and *Nocardia* infections after bronchial thermoplasty

**DOI:** 10.1002/rcr2.392

**Published:** 2018-11-28

**Authors:** Sachi Matsubayashi, Motoyasu Iikura, Takanori Numata, Shinyu Izumi, Haruhito Sugiyama

**Affiliations:** ^1^ Department of Respiratory Medicine National Center for Global Health and Medicine Tokyo Japan; ^2^ Department of Respiratory Medicine Jikei University School of Medicine Tokyo Japan

**Keywords:** *Aspergillus*, bronchial thermoplasty, *Nocardia*, severe asthma

## Abstract

Bronchial thermoplasty (BT) is a bronchoscopic treatment for severe asthma. A 35‐year‐old woman with uncontrolled severe asthma despite maximal pharmacological treatment underwent BT and started coughing after the first procedure. One month later, during the second BT procedure, there were white ulcerous lesions on the right B9 bronchus. Culture of the bronchial brushing specimen showed *Aspergillus fumigatus*, for which voriconazole was started for treatment. On the third BT procedure, endobronchial mucus sampling demonstrated *Nocardia* spp., for which trimethoprim‐sulfamethoxazole was given for three months. Seven months after the third BT procedure, no particular endobronchial lesions were found, and no abnormal pathogens were obtained by culture. The resulting bronchial infection in this case may be attributed to the use of systemic steroids, which rendered the patient immunocompromised, and to tissue fragility that was caused by the thermal energy from the BT procedure. Culture of endobronchial mucus should be considered during BT.

## Introduction

Bronchial thermoplasty (BT) is a bronchoscopic procedure that uses thermal energy to reduce airway smooth muscle mass for treating uncontrolled severe asthma. Bronchial oedema and radiological changes are generally known as the major complications of BT, but infections have rarely been reported. We describe a case of fungal and bacterial infections that developed after BT.

## Case Report

A 35‐year‐old woman was treated for severe asthma with high‐dose inhaled corticosteroid and long‐acting beta‐2 agonist, along with omalizumab. However, she continued to experience frequent asthma exacerbations despite regular systemic steroid use. Her asthmatic symptoms started when she was 2 years old. She required hospitalization several times a year during childhood and in her 20s because of asthma exacerbation, and then, she started taking omalizumab. Because of poorly controlled symptoms despite maximal pharmacological treatment, she was admitted to our hospital for BT. Her chest exam demonstrated wheeze only during forced expiration but no crackles. No other abnormalities were observed by physical examination. She is allergic to cedar pollen and has allergic rhinitis.

Blood testing showed IgE of 109 U/mL, *Aspergillus*‐specific IgE was 20.9 UA/mL, and the white blood cell count was 7790/μL, including 2570/μL of lymphocyte without any eosinophils; at this time, she was taking 4 mg of prednisolone and omalizumab. She took 32 mg/day of methylprednisolone from three days before to the next day of the BT procedure. Her lymphocyte count dropped to 426/μL the day before the procedure.

**Figure 1:**
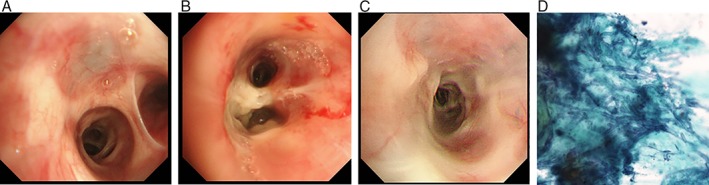
Findings during the bronchial thermoplasty (BT) procedures on the right B9 bronchus. Bronchoscopic images at (A) first BT, (B) second BT, and (C) third BT. (D) Photomicrograph of the bronchial brushing specimen during the second BT shows the presence of Y‐shaped hyphae (Papanicolaou stain, 40× magnification).

Chest computed tomography (CT) at the expiratory phase showed scattered areas of air trapping, but there was no thickening or dilatation of the bronchial walls or mucus plugging. Sinus CT demonstrated no evidence of chronic sinusitis (data not shown). The first BT procedure was completed properly. The bronchial epithelium was oedematous and easy to bleed, but there were no ulcers or purulent mucus (Fig. 1A). The total number of activations on the right lower bronchi was 81. No abnormal pathogens were cultured from the bronchial mucus.

The second BT procedure was conducted after one month. White ulcerous lesions were found on the right B9 bronchus, where the first BT procedure was performed (Fig. 1B). *Aspergillus fumigatus* with neutrophilic, and eosinophilic bronchial inflammation was detected from the bronchial brushing at the right B9 bronchus (Fig. 1D). Therefore, she was given voriconazole for seven months for the treatment of bronchial aspergillosis. Three months after the second BT procedure, a third BT procedure was performed (Fig. 1C); culture of endobronchial mucus at this time demonstrated *Nocardia* spp., for which she was prescribed trimethoprim‐sulfamethoxazole for three months.

**Figure 2:**
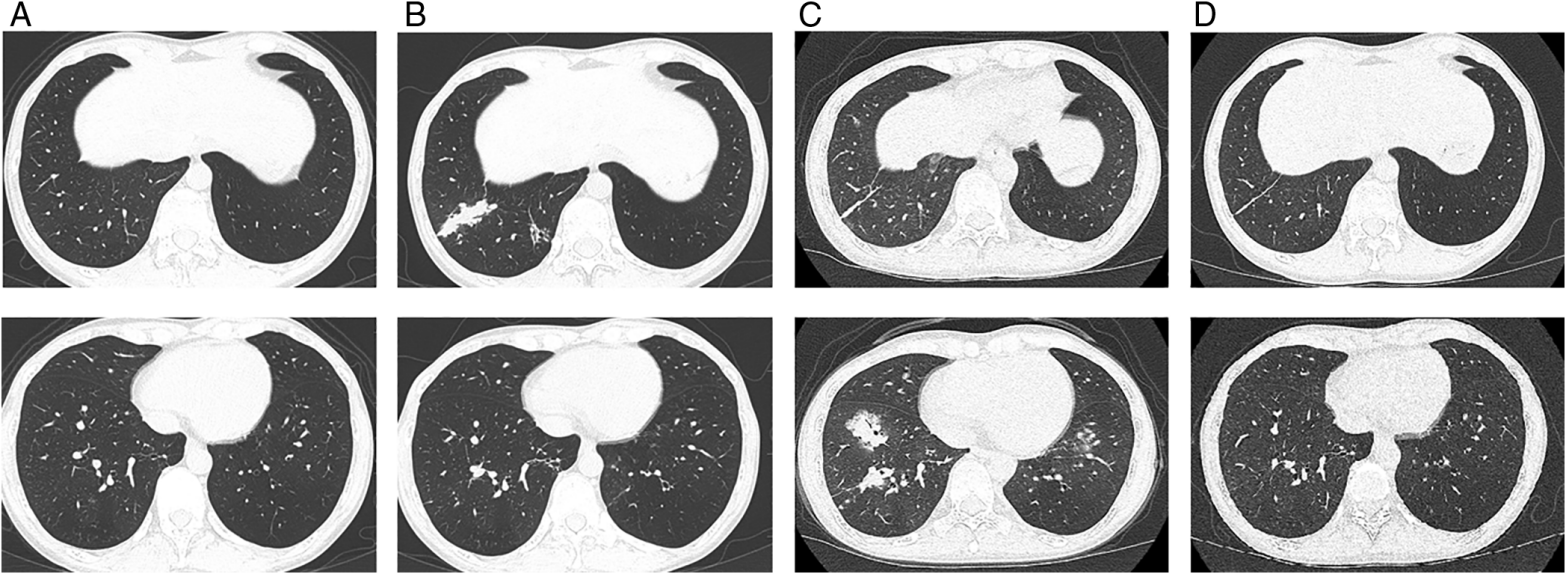
Chest computed tomography images before and after bronchial thermoplasty (BT). The images in the upper section are slices around the right segment B9, and the images in the lower section are slices around right lower lobe segments (A) before BT and after BT completion at (B) 1 month, (C) 7 months, and (D) 12 months.

Chest CT at one month after the third BT procedure showed partial consolidation around the right B9 bronchus at the location of the endobronchial aspergillosis (Fig. 2B). Seven months after the last BT procedure, CT showed resolution of the right B9 occlusion, but there was a new consolidation around the right B8 bronchus (Fig. 2C). Pathological examination of the transbronchial biopsy from this lesion showed obstructive bronchiolitis with eosinophilic infiltration, without abnormal pathogen on culture. This consolidation resolved one year after the last BT (Fig. 2D).

## Discussion

Previous studies have evaluated and validated the efficacy, feasibility, and safety of BT for severe asthma patients. One of the definitive studies on BT was the Asthma Intervention Research (AIR2) study, which showed that BT improved asthma‐related Quality of Life (QOL) and reduced the frequency of severe exacerbations compared to a sham‐controlled group 1. Moreover, this efficacy of BT could be sustained for at least five years 2. We also reported that BT improved the QOL, exacerbations, symptoms, and obstructive lung function of Japanese asthmatic patients, with few adverse events 3.

However, some complications associated with BT have been reported. Burn et al. pointed out that their BT patients experienced adverse events more frequently than described in previous clinical trials 4. Bronchial oedema and radiological changes are generally known as major complications of BT. A recent study showed that BT induces an epithelial sloughing in the acute phase 5. In the AIR2 trial, one patient in the BT group needed hospitalization due to lower respiratory tract infection 1; however, no other information about the infection was shown. To the best of our knowledge, the present case was the first to show the particular complications of bronchial infection after BT.

Because the patient was taking systemic corticosteroids regularly to control asthmatic attacks and was also on high‐dose systemic steroids around the time of the BT procedure, she might have been immunocompromised. In addition, the thermal energy from BT might have cause tissue fragility, which predisposed to bronchial infection. We could not confirm the relationships between these infections and high‐dose steroid use, or BT procedure, but we suggest these relationships because *Aspergillus* was cultured where we did the first BT procedure. There are no reports that mention the cultures of endobronchial mucus during and after BT, but bronchial specimens should be collected for early detection of lower respiratory infection around BT procedure.

High titre of *Aspergillus*‐specific IgE suggested the possibility of allergic bronchopulmonary aspergillosis (ABPA). However, there were no fleeting parenchymal opacities or bronchiectasis on the CT images before the BT procedures. Moreover, there was no evidence of chronic sinusitis or mucus plugging. Although we did not perform precipitating antibody or skin test, the diagnosis of ABPA was unlikely.

In conclusion, we reported a case of fungal and bacterial infections that developed after BT. The possibility of bronchial infection after BT should be considered, and endobronchial mucus sampling for culture is recommended during BT.

### Disclosure Statement

Appropriate written informed consent was obtained for publication of this case report and accompanying images.
